# Metabolic Rewiring of *Mycobacterium tuberculosis* upon Drug Treatment and Antibiotics Resistance

**DOI:** 10.3390/metabo14010063

**Published:** 2024-01-18

**Authors:** Biplab Singha, Sumit Murmu, Tripti Nair, Rahul Singh Rawat, Aditya Kumar Sharma, Vijay Soni

**Affiliations:** 1 Department of Microbiology and Physiological Systems, University of Massachusetts Chan Medical School, Worcester, MA 01605, USA; biplab.singha1@umassmed.edu; 2 Regional Centre of Biotechnology, Faridabad 121001, India; sumit.murmu@rcb.res.in; 3 Leonard Davis School of Gerontology, University of Southern California, Los Angeles, CA 90089, USA; tnair@usc.edu; 4 Eukaryotic Gene Expression Laboratory, National Institute of Immunology, New Delhi 110067, India; rahulsingh@nii.ac.in; 5 Department of Pathology, College of Medicine, University of Illinois at Chicago, Chicago, IL 60612, USA; 6 Division of Infectious Diseases, Weill Department of Medicine, Weill Cornell Medicine, New York, NY 10021, USA

**Keywords:** *Mycobacterium tuberculosis*, tuberculosis, antimicrobial resistance, metabolic rewiring, anti-TB drugs, drug resistance, metabolomics, metabolism

## Abstract

Tuberculosis (TB), caused by *Mycobacterium tuberculosis* (*Mtb*), remains a significant global health challenge, further compounded by the issue of antimicrobial resistance (AMR). AMR is a result of several system-level molecular rearrangements enabling bacteria to evolve with better survival capacities: metabolic rewiring is one of them. In this review, we present a detailed analysis of the metabolic rewiring of *Mtb* in response to anti-TB drugs and elucidate the dynamic mechanisms of bacterial metabolism contributing to drug efficacy and resistance. We have discussed the current state of AMR, its role in the prevalence of the disease, and the limitations of current anti-TB drug regimens. Further, the concept of metabolic rewiring is defined, underscoring its relevance in understanding drug resistance and the biotransformation of drugs by *Mtb*. The review proceeds to discuss the metabolic adaptations of *Mtb* to drug treatment, and the pleiotropic effects of anti-TB drugs on *Mtb* metabolism. Next, the association between metabolic changes and antimycobacterial resistance, including intrinsic and acquired drug resistance, is discussed. The review concludes by summarizing the challenges of anti-TB treatment from a metabolic viewpoint, justifying the need for this discussion in the context of novel drug discovery, repositioning, and repurposing to control AMR in TB.

## 1. Introduction

The World Health Organization (WHO) estimates that tuberculosis (TB) will regain its position as the leading cause of death due to an infectious agent in the world after briefly losing its title during the COVID-19 pandemic [1]. TB is an airborne lung disease caused by the bacillus *Mycobacterium tuberculosis* (*Mtb*). It can also infect other organs and tissues such as spine, lymph nodes, kidneys, and brain, and known as extrapulmonary TB (EPTB). Besides active-TB (ATB), latent TB infection (LTBI), where *Mtb* remains dormant for decades without causing the disease, also accounts for a major percentage of infections.

TB treatment typically involves a combination of antibiotics, initially using a standard regimen of isoniazid, rifampicin, pyrazinamide, and ethambutol (HRZE) for 2 months to prevent drug resistance, followed by a 4- to 6-month continuation phase with isoniazid and rifampicin (HR). In cases of multi-drug-resistant TB (MDR-TB), a more complex longer treatment involving second-line drugs like fluoroquinolones and injectables such as amikacin, kanamycin, or capreomycin is necessary. For extensively drug-resistant TB (XDR-TB), the regimen becomes even more challenging, requiring a combination of second-line drugs and newer agents like bedaquiline and linezolid. However, treatment can fail for various reasons. The primary factors contributing to the failure of drug treatments include drug resistance due to improper antibiotic use, patient non-adherence to treatment regimens, often caused by side effects or socio-economic factors, and pharmacokinetic variability affecting the drug efficacy. HIV co-infection presents additional challenges due to interactions between TB drugs and antiretroviral therapy and a compromised immune system. Advanced TB can lead to extensive lung damage, hindering effective drug delivery, while serious side effects like hepatotoxicity and optic neuritis can lead to the discontinuation of treatment. Socio-economic issues such as poverty and inadequate healthcare access further impede treatment effectiveness. Strategies like Directly Observed Therapy (DOT) and the development of new TB drugs and diagnostic methods are essential in addressing these challenges and improving TB treatment management.

Different bacteria, including *Mtb*, have system-level adaptability, helping microorganisms to sustain themselves in non-viable conditions such as under stresses and drug treatments. The role of the genome, transcriptome, and proteome has been discussed in different studies, but a comprehensive discussion is due on “how *Mtb* utilizes metabolic pathways as a tool to counteract antibiotic treatments for survival”. Metabolic alterations are not merely a passive response to the stress imposed by antibiotics but represent a sophisticated adaptation strategy that enables the bacteria to survive and proliferate in the hostile environment created by antimicrobial agents. Considering all these facts, in this article, we review how these biochemical adaptations contribute to drug resistance, their role in TB treatment, and potential avenues for overcoming the challenges of AMR. In examining the complex interaction between *Mtb*’s metabolism and drug exposure, this review article intends to provide insight into *Mtb* survivorship and resistance, paving the way for new therapeutic interventions to combat this ancient and constantly evolving disease.

## 2. Anti-Mycobacterial Drugs and Their Targets

TB is a potent public health challenge and has been treated using a wide array of drugs and treatment strategies over the years across countries and continents. The combination and duration of the treatment and dosage of medication rely on the active or latent condition of TB. The anti-TB medications rifampin, ethambutol, isoniazid, and pyrazinamide fall within the first line of FDA-approved drugs. However, the common prognosis of TB is the development of multi-drug-resistance to the first-line medications rifampicin and isoniazid. In the follow-up, the second-line MDR-TB treatments consist of kanamycin, amikacin, and capreomycin injections. Fluoroquinolones such as moxifloxacin, levofloxacin, and gatifloxacin are also among the common second-line agents. The recent array of FDA-approved drugs for MDR-TB is pretomanid used in combination with linezolid and bedaquiline. The most recent challenge is the rising number of XDR-TB cases displaying resistance to first-line treatments like rifampin and isoniazid, along with resistance toward one of the second-line treatments aminoglycoside, and to a fluoroquinolone (Table 1). 

### Antibiotics and Their Mode of Actions

The most used anti-TB drugs are rifampin, isoniazid, pyrazinamide, ethambutol, aminoglycosides, and fluoroquinolones. Rifampin and its derivatives inhibit DNA-dependent RNA polymerase, further inhibiting bacterial transcription and protein synthesis. Isoniazid blocks the synthesis of mycolic acid. Pyrazinamide in its active form, pyrazinoic acid, perturbs trans-translation. Ethambutol affects arabinosyltransferases, thus preventing the synthesis of the mycobacterial cell walls. Aminoglycosides (streptomycin, kanamycin, and amikacin) stop protein synthesis by binding to the 30S subunit of ribosomes. Meanwhile, fluoroquinolones (levofloxacin, moxifloxacin, and gatifloxacin) hinder DNA gyrase and topoisomerase IV activity, further inhibiting DNA synthesis. *Mtb* undergoes spontaneous mutations, leading to multidrug resistance and the eventual recurrence of the pathologies and symptoms associated with TB. The conventional anti-TB regimens sometimes do not target *Mtb* under certain acidic hypoxic conditions of macrophages. In the last couple of decades, ongoing efforts to understand the molecular mechanisms of anti-TB resistance have taken the forefront.

**Figure 1:**
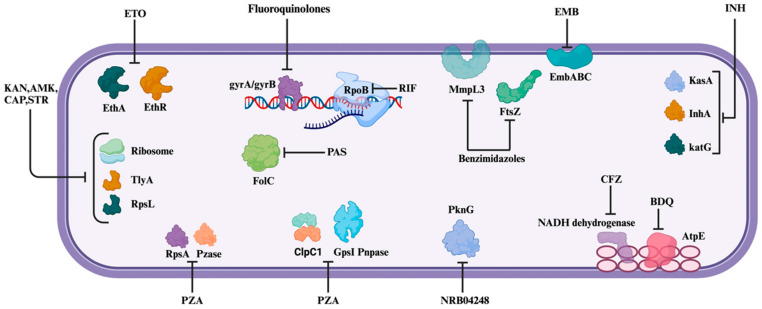
Mode of action of different anti-TB drugs—the schematic diagram depicts the cellular target of various anti-TB drugs such as isoniazid (INH), ethambutol (EMB), amikacin (AMK), kanamycin (KAN), pyrazinamide (PZA), ethionamide (ETO), streptomycin (STR), capreomycin (CAP), para-minosalicylic acid (PAS), and clofazimine (CFZ).

Resistance to first-line drugs is commonly observed after the antibiotic phase. Isoniazid resistance is associated with alterations in the catalase-peroxidase gene (*katG*) and the mycolic acid synthesis gene (*inhA*). Resistance to rifampin and its derivatives (rifabutin, rifapentine, and rifalazil) is associated with mutations in the RNA polymerase subunit B (*rpoB*). Elimination of the pyrazinamidase/nicotinamidase activity via mutations in the gene *pncA* leads to pyrazinamide resistance. Ethambutol resistance arises due to genetic mutations within the *embABC* operon, which correspondingly facilitates arabinosyl transferase production. Second-line drugs also face challenges associated with resistance due to aberrant genetic mutations. Streptomycin resistance is associated with mutations in the ribosomal S12 protein 16S rRNA gene. Capreomycin resistance is due to mutagenesis of the *tlyA* gene (homologous to rRNA methyltransferases). Quinolone resistance corresponds with mutations in the DNA gyrase gene. Ethionamide resistance is linked to *inhA* mutations. The resistance to PAS is observed due to mutations within the gene encoding thymidylate synthase A (Figure 1, Table 2). Table 2 briefly describes the mode of action and resistance displayed by *Mtb* to a prevalent array of drugs.

Advances in *Mtb* drug development encompass the exploration of genome-wide sequences and the identification of the key signaling and associated metabolic pathways. In this strenuous approach, the chances of predicting the novel candidate with high specificity, low toxicity, and low efficacy are low. In the meantime, the reengineering and repositioning of known drugs are sought after to achieve better results in a time-efficient manner. However, the chances of pre-existing resistance still threaten this goal. Therefore, discovering novel targets and approaches (compounds) remains at the forefront to counter. 

Expanding the existing drugs toolkit by developing upon previously available drugs is a common approach to combating drug resistance but is substantially unsuccessful in *Mtb* due to its intrinsic features. To combat MDR-TB, drug repurposing or drug repositioning is utilized as an effective strategy. Drug repurposing involves the use of a pre-existing drug that has been approved or is in circulation against specific diseases or symptoms but also shows favorable effects against other diseases. A classic example of this is Sildenafil, which was originally manufactured as an anti-hypertension drug but was also found to be an effective inhibitor of phosphodiesters-5 to treat erectile dysfunction [54]. Drug repurposing has attracted the attention of many pharmaceutical companies due to the various advantages, like reduced risk, shortened development time, faster approvals for clinical trials, fewer economic challenges, and less skepticism in acceptance. The emergence of drug-resistant TB has forced scientists to repurpose existing drugs to combat the pathogen. A few of the existing drugs to prevent the TB are listed in Table 3.

## 3. Mycobacterial Metabolic Rewiring upon Drug Treatment

*Mtb* undergoes metabolic rewiring to adapt to various host environments’ stresses during infection. It involves a shift in metabolic pathways and nutrient utilization to ensure survival and persistence inside the host. Upon drug-induced stress, *Mtb* can modulate itself using transcriptional and post-translational gene regulation, metabolic slowdown, metabolic shifting, cell wall thickening, the upregulation of efflux pumps, and genetic adaptations conferring tolerance toward known anti-mycobacterial drugs [76]. Understanding these adaptations is crucial for developing effective TB treatments. The survival of mycobacteria upon drug-induced stress strongly depends on their ability to modulate their metabolic pathways, commonly known as metabolic rewiring. Several publications in the literature reveal that interconnected metabolic pathways are involved in the establishment of a drug-tolerant state [77]. While some mechanisms of tolerance are nonspecific and occur upon exposure across different types of stress, in this section, we will discuss changes in metabolic state upon drug-induced stress which occur only following exposure to one specific drug or drug class (drug-specific metabolic rewiring) (Table 4).

### 3.1. Metabolic Rewiring of Mtb in Drug-Susceptible TB

Drug-susceptible *Mtb* is affected by the first-line drugs of the TB regimen that includes isoniazid (INH), rifampicin (RIF), pyrazinamide (PZA), and ethambutol (EMB) [86]. Isoniazid is a prodrug that is converted by *Mtb* intracellular catalase-peroxidase (KatG) into isonicotinoyl, which binds with NAD+ to form an adduct, isonicotinoyl–NAD (INH-NAD+). The KatG-mediated INH conversion produces a large amount of mutagenic ROS, which contribute to the strong bactericidal activity of INH [112]. The transcriptional response to INH stress begins immediately after INH exposure [113,114,115], with over 100 genes activated in the bacilli that survive the first INH death [77,116]. Most INH-regulated genes are found in the metabolic pathways involved in lipid metabolism and cell wall synthesis, reflecting the two main INH tolerance mechanisms: a reduced need for lipid synthesis due to decreased growth and metabolic rewiring of the lipid metabolism and redox homeostasis pathways to avoid INH-induced disruption of the cell wall integrity. Under INH stress, the redox equilibrium is disrupted due to altered lipid metabolism and decreased NADH synthesis in the TCA cycle, resulting in NAD+ build-up. To reduce the NAD+ generation under INH pressure, *Mtb* downregulates the expression of NADH dehydrogenase-encoding genes. The chemical balance moves toward lower NAD+-to-isonicotinoyl binding, which reduces the InhA inhibition [117,118]. INH also increases the TCA cycle’s glyoxylate bypass by upregulating isocitrate lyase, resulting in lower rates of amino acid precursor turnover and fewer high-energy reducing agents like NADH, which likely results in lower metabolic activity and ROS production [119]. The sigma factors SigB and SigE were also found to have a role in the metabolic rewiring of *Mtb* upon INH treatment. *M. smegmatis* has been examined, where SigB interacts with RNA polymerase binding protein A (RbpA) to increase the transcription of polyphosphate kinase 1 (*ppk1*). Increased Ppk1 activity causes polyphosphate build-up, which has been linked to tolerance in response to heavy metal or osmotic stress in *E. coli*, Lactobacillus, and other bacteria [80,120,121]. Research has shown that *Mtb* can cause metabolic rewiring, the mistranslation of *rpoB*, the overexpression of *rpoB*, and enhanced efflux pump activity to establish tolerance to RIF exposure [81,82,83]. A recent metabolomics study showed a total of 173 metabolites, associated with the purine, pyrimidine, arginine, tyrosine, phenylalanine, and tryptophan metabolic pathways and tricarboxylic acid cycle metabolite, arginine, and phosphoenolpyruvate metabolism, were significantly altered by rifampicin [79]. In addition, RIF can induce metabolic slowness through reduced replication in *Mtb*, as RIF kills more in the log phase as compared to the stationary phase [84]. PZA-induced metabolic rewiring in *Mtb* has not been studied yet. PZA is only effective against *Mtb* bacteria that are stationary and have slowed down metabolically when they are still under the influence of INH and RIF. This discovery may have an explanation related to the energy-dependent method according to which the active form of PZA, which is pyrazinoic acid (POA), is pumped out of *Mtb* via an efflux pump [87]. Although there is little information on EMB tolerance, one study discovered that EMB is very bactericidal when *Mtb* does not have the SigB or SigE sigma factors, indicating that the sigma factors SigB and SigE may cause tolerance when exposed to EMB [93].

### 3.2. Metabolic Rewiring of Mtb in MDR and XDR Strains

The metabolic rewiring of *Mtb* in response to the drugs used for treating MDR and XDR-TB infection is an ongoing area of research. Recent reports suggest only genetic mutations in the *gyrA*, *gyrB*, *Rv2686c*, *Rv2687c*, and *Rv2688c* genes, which confer resistance to moxifloxacin, a fluoroquinolone [94], and mutations in 13 new genes along with *alr* (*Rv3423c*), encoding alanine racemase, confer resistance to D-cycloserine in *Mtb* [96]. However, the metabolic rewiring of *Mtb* upon bedaquiline (BDQ) treatment leads to an increase in 7 of the 10 universal stress proteins in *Mtb* that are known to exist. Furthermore, by triggering a BDQ stress response, the overexpression of several transcription factors—including SigG, Rv0324, and Rv0880—creates BDQ tolerance [100,101]. Upon ethionamide (ETH) treatment, *Mtb* elicits a transcriptional response comparable to that seen in INH stress, which is not unexpected since both drugs target mycolic acid production. A total of 70 genes were differentially expressed upon ETH-induced stress; out of these, 39 genes behaved similarly to INH-induced stress. Of the genes that are specifically induced by ETH, 15 were associated with lipid metabolism, and 18 were associated with cell wall synthesis and cell processes [104]. Amikacin, an aminoglycoside, upon pressure, causes *Mtb* to overexpress *whiB7*, which impacts genes related to ribosomal protection (*eis*) and drug efflux (*tap*), hence decreasing the susceptibility to almost all the aminoglycosides used in TB therapy, including amikacin [108,110]. Unlike the irreversible mutations that provide resistance to amikacin, kanamycin, and capreomycin in the *eis* promoter regions, the overexpression of *whiB7* and its upregulation can lead to a transient reduction in aminoglycoside sensitivity [122,123]. Given that it binds to WhiB7 to create a WhiB7–SigA promoter complex, SigA could be involved in this mechanism. Additionally, SigA interacts with the DevR/DosR dormancy regulon, causing changes in the metabolism of carbon, energy, and lipids, as well as lower metabolic rates and growth arrest [120,124].

Since genetic mutations are usually permanent, transient frameshift mutations and changes in metabolic processes are the first steps that *Mtb* can initiate to tolerate drug-induced stress. Frameshift mutations in the *glpK* gene, which produces the glycerol-3-kinase, have been linked, in vitro and in clinical isolates, to resistance to INH, RIF, PZA, and moxifloxacin. The drug effectiveness may be affected by *glpK* frameshift mutations that reduce growth, alter the cellular structure, and promote the expression of stress response regulators (such as DosR and SigH) [125,126]. Multiple metabolic networks within *Mtb*, which are related to central carbon metabolism and carbon fluxes, are rewired to reduce the drug-induced stress [127]. A recently published article showed the role of cAMP signaling in intrinsic drug resistance, which unravels a whole new avenue of research in this area [128].

## 4. Association of Metabolic Changes with Antimycobacterial Resistance

### 4.1. Intrinsic Drug Resistance in Mtb and Metabolic Determinants

*Mtb* has exhibited a higher level of intrinsic resistance to various antibiotics [129,130]. The mycobacterial envelope is the primary factor that contributes to this intrinsic resistance. The main components of the mycobacterial envelope are mycolic acids, arabinogalactan, and peptidoglycan [129]. Peptidoglycan is the inside layer and is nearest to the plasma membrane. It has a covalent attachment to arabinogalactan polymers [129]. The long-chain fatty acids known as mycolic acids, which are connected to arabinogalactan polymers, create a mycobacterial outer membrane, a bilayer of the whole outer membrane [130,131]. This arrangement of peptidoglycan, arabinogalactan, mycolic acids, and lipid membranes leads to the formation of an impermeable outer membrane that prevents the entry of various classes of antibiotics, contributing to intrinsic resistance to antibiotics [130,131]. It is known that small molecules of a hydrophilic nature are unable to penetrate the mycobacterial outer membrane and are suggested to depend upon porin-like protein for entry [130,132]. However, hydrophobic compounds become stuck in the mycolic acid and fail to pass the mycobacterial outer membrane. Because of this phenomenon, ethambutol, a drug that stops arabinogalactan and lipoarabinogalactan synthesis, is included as part of a first-line regimen for TB [129]. Ethambutol inhibits arabinogalactan and lipoarabinogalactan biosynthesis [133], which allows other antibiotics like rifampicin, isoniazid, and pyrazinamide to pass into the bacteria [129]. Recently, studies have suggested that ethambutol is responsible for the effective distribution of drugs in the lungs [134]. Furthermore, ethambutol and rifampicin combine to form a synergistic partnership that facilitates the passage of hydrophobic, high-molecular-weight rifampicin through the mycobacterial envelope, acting as a barrier [135]. Similarly, bedaquiline’s action is increased upon the knockdown of many enzymes involved in the biosynthesis of arabinogalactan and mycolic acid [136,137]. The combination of bedaquiline with other drugs that inhibit the peptidoglycan–araninogalactan–lipoarabinogalactan complex increases the efficacy of bedaquiline [136,137].

Another factor that contributes to the intrinsic drug resistance is efflux pumps. These transmembrane proteins assist in transporting drugs from the inside to the outside, which decreases the drug effectiveness [138]. *Mtb* harbors numerous drug efflux pumps, with proton-driven efflux pumps representing a prominent group [139]. The activity of other pumps is controlled by the breakdown of ATP and the exchange of drugs for protons [139,140]. Studies have shown that the MmpS5/L5 efflux pump is active in the efflux of drugs such as bedaquiline and clofazimine [141]. Similarly, studies have demonstrated that Rv1258c is an efflux pump that is active against numerous antitubercular medications, such as rifampicin and streptomycin [142,143]. Moreover, the role of efflux pump inhibitors is shown to be effective in the inhibition of mycobacterial survival [139]. Apart from this, many transcription factors like WhiB7 regulate the transcription of other resistance factors during drug treatment and metabolic changes, namely, *eis,* an aminoglycoside acetyltransferase [144]. Studies have indicated that WhiB7 controls *erm* (37), a ribosomal RNA methyltransferase that alters the macrolide binding site on 23S rRNA, which inhibits drug binding [145]. Various aminoglycoside/cyclic peptide antibiotics also undergo acetylation by the Eis protein. Studies have shown that the Eis protein acetylates to inactivate capreomycin, which helps the mycobacteria resist antibiotic regimens [123,146]. Mycobacteria also develop intrinsic resistance using enzymes that cause drug inactivation or drug modifications. The use of β-lactamases enzymes, which hydrolyze the β-lactam ring of the antibiotics, helps mycobacteria survive. *Mtb* encodes BlaC, a class A β-lactamase, which has a broad substrate specificity with varying affinities. However, BlaC is inhibited by clavulanate, a β-lactamase inhibitor [147].

### 4.2. Acquired Drug Resistance: A Microbial Metabolic Catastrophe

Antimicrobial resistance is a phenomenon that occurs when microbes undergo genetic changes that allow them to resist the effects of drugs. However, the overuse and misuse of drugs have accelerated the emergence and spread of resistance, making it a huge problem. One way in which microbes can acquire resistance is through changes in metabolic pathways, making them less susceptible to drugs [148]. *Mtb* can develop alternative metabolic pathways that bypass the target of drugs. Chromosomal mutations also lead to the development of drug resistance through a variety of mechanisms, namely the induction of efflux pumps, target overexpression, drug target modifications, and the abrogation of prodrug activation. The interactions between drugs and drug targets are highly specific. Any alteration in these interaction sites can make *Mtb* resistant to antibiotics [149]. In *Mtb*, altered drug susceptibility regularly arises from a non-synonymous mutation in genes encoding drug targets or nucleotide substitutions within the operon responsible for ribosomal RNA production. For example, mutations in the QRDR region of *gyrA* and *gyrB* are responsible for quinolone resistance [150]. Studies have shown that 7 mutations in *gyrA* and 19 mutations in *gyrB* are responsible for fluoroquinolone resistance [151]. Mutations within the rifampicin resistance-determining region, an active site of DNA-dependent RNA polymerase, lead to diminished binding of rifampicin to its targets, ultimately conferring resistance in *Mtb* [78].

Several antimycobacterial drugs, including isoniazid, pyrazinamide, ethionamide, para-aminosalicylic acid, delamanid, and pretomanid, exist as prodrugs and require specific enzymes for their activation. For example, pyrazinamide is a prodrug that is converted into pyrazinoic acid by pyrazinamidase/nicotinamidase (PncA) to inhibit mycolic acid synthesis, which causes *Mtb* death [87]. However, *Mtb* acquires mutations in the PncA gene that prevent the conversion of pyrazinamide into pyrazinoic acid, so *Mtb* become less susceptible to pyrazinamide [87]. Similarly, once isoniazid enters the *Mtb* cell, isoniazid is converted into isonicotinoyl hydrazine by the enzyme catalase-peroxidase (KatG), and then the active form of isoniazid inhibits the enzyme InhA, which is a vital enzyme for mycolic acid synthesis [152]. An alternative mechanism of acquired drug resistance involves mutations in the repressors or promoters of the drug target gene, leading to the overexpression of the drug target protein. For example, isoniazid’s efficacy can be reduced by the overexpression of its drug target [153]. This acquired resistance can normally be countered by increasing the isoniazid dosage. Cycloserine’s effectiveness can also be hampered by the overexpression of its drug target, leading to acquired resistance. However, unlike isoniazid, a cycloserine dosage increase cannot overcome this acquired resistance due to its adverse effects at higher concentrations [154].

The evolution of MDR-TB and XDR-TB strains is a result of many metabolic and genetic rearrangements. It can affect the growth rate, division cycle, virulence, and response to other treatments. How AMR pushes the boundaries and rewires the metabolism upon different drug treatments or similar drugs aiming at other sites of the same target protein is a matter of further research. Since the development of AMR is a process of adaptation and survival, studying such interactions can help to develop potent strategies to control its spread.

## 5. Prospects of Metabolic Rewiring of *Mtb* in Novel Therapeutic Development

The abuse and misuse of antibiotics is the leading impetus of the AMR in *Mtb* and has currently become one of the most challenging healthcare problems in the world. Unlike AMR in other pathogenic bacterial species, the AMR in *Mtb* draws serious attention due to its genomic characteristics. The genome of *Mtb* (size of 4.4 Mb) is characterized by low mutations, and still there is not a shred of evidence of HGT (horizontal gene transfer) in the strains to confer the AMR [155]. The lack of HGT, combined with a lower mutation rate and an obligate pathogen, make *Mtb* a challenging target for which to develop new drugs against AMR *Mtb*.

Metabolic regulation in *Mtb* is also one of the key factors that drives AMR in the bacteria. Most of the drug-based clinical research undertaken on *Mtb* is under nutrient-rich conditions in the laboratory, whereas in vivo, the *Mtb* grows under nutrient-limiting conditions; hence, the metabolism is quite contrasting [156,157]. During in vivo conditions, *Mtb* slows down its metabolism by rerouting pathways from energy generation to energy storage and reducing growth. These metabolic changes induce heterogeneity in the bacterial community, and hence their susceptibility to drugs changes stochastically. A classic example is during stress conditions, *Mtb* redirects its metabolite from the TCA (tricarboxylic acid) cycle to TAG (triacylglycerol) synthesis [158]. Slowing down the TCA cycle results in reduced production of catabolism enzymes, reduced amino acid synthesis, and lower protein translation and increases the production of certain enzymes that enhance the resistance of the *Mtb* against INH, RIF, and STR [119]. The accumulation of enzymes and metabolites like methylcitrate intermediates and phosphoenolpyruvates during hypoxia confers tolerance to INH and BDQ [159,160]. In another example, *Mtb* utilizes trehalose for carbohydrate storage and as a component in the cell surface glycolipids. Under hypoxic conditions, *Mtb* downregulates certain glycolipids and redirects trehalose toward the biosynthesis of central carbon metabolism (CCM) intermediates. In a biofilm model, the trehalose metabolism shifts toward CCM intermediates, which is associated with an increase in drug tolerance. *Mtb* trehalose deletion mutants were unable to make this shift during hypoxic conditions and exhibited rapid ATP depletion and increased susceptibility to the drug BDQ [161].

### 5.1. Strategies to Combat Mtb Metabolic Rewiring

The metabolic pathway in *Mtb* is quite complex and flexible as it can survive various environmental and antibiotic stresses and in diverse in vivo conditions. The metabolic flexibility of *Mtb* and its metabolic rewiring during different nutritional stresses make it somewhat of a difficult target for treatment. Some of the key metabolic pathways that impart the bacteria its robustness are discussed below. Understanding these pathways is key in targeting specific areas that can lead to the development of efficient drugs to cure AMR-TB.

### 5.2. Nucleotide Metabolism

The nucleotide metabolism is a key pathway that serves in bacterial DNA replication and repair and has been explored extensively [162]. RNRs (ribonuclease reductases) are particularly of interest as drug targets that maintain the homeostasis of the dNTP pool level in the bacteria [163], but due to the presence of multiple redundant RNRs, they have become unattractive targets for drug development [164]. Out of many RNRs, NrdEF2 is a class Ib RNR that is an essential growth regulator under oxidative stress. The inactivation of this regulator triggers the activation of the *nrdF2* and *nrdH* genes, which make up a glutaredoxin that reduces NrdF2. NrdF2 maintains the dNTP pool levels during DNA synthesis under oxidative stress and facilitates the survival of bacteria under stress. Phenotypic screening of drugs against *Mtb* led to the identification of a potent molecule belonging to the homopiperazine (Fasudil) scaffold, which is a known inhibitor of Rho-associated protein kinase. A key target of the inhibitory compound in *Mtb* was found to be GuaB2 protein, which is a member of the inosine 5′-monophosphate dehydrogenase (IMPDH) family [165]. IMPDH converts inosine 5′-monophosphate into Xanthosine monophosphate, which is the initial step involved in the biosynthesis of guanine nucleotide. Guanine nucleotides are involved in multiple biosynthetic pathways in *Mtb* like nucleic acid synthesis, protein synthesis, cell wall biogenesis, etc., and the inhibition of guanine nucleotide synthesis by restricting the inosine 5′-monophosphate to xanthosine monophosphate is found to be lethal for *Mtb* [165]. To assess GuaB2 as a drug target, GuaB2 was depleted using gene silencing or inhibition with drugs, and it was found that GuaB2 is essential for bacterial survival in the host, and rescue from lethality in a GuaB2 mutant requires a high external concentration of guanine (>100 μM). Doxycyclin-fed mice with silenced GuaB2 experienced a complete halt in the replication of *Mtb,* which resulted in faster clearance.

### 5.3. Amino Acid Biosynthesis

The *Mtb* amino acid biosynthesis pathways are generally non-essential during infection because they can scavenge nutrients from the host. However, the variation and flexibility in the *Mtb* metabolic pathway and the advancement of genetic validation techniques have shown them to be conditionally essential, i.e., necessary in vivo even when dispensable in vitro. Notably, the deletion or inhibition of enzymes involved in tryptophan, histidine, and methionine biosynthesis leads to *Mtb* cell death in vitro, whereas proline and leucine produce only a static response, and their absence is tolerated [166,167]. The in vivo scenario is even more intricate, with *Mtb* scavenging nutrients from the host cells, and varying nutrient availability between tissues and different parts of the granuloma. Despite some amino acid availability in vivo, both tryptophan and branched-chain amino acid auxotrophs exhibit significant attenuation in mice [168,169]. Importantly, these pathways lack human homologs, suggesting their potential as promising therapeutic targets. The importance of tryptophan biosynthesis for *Mtb* in both in vitro and in vivo conditions has been well established [166]. During *Mtb* infection, the CD4 T-cells produce interferon-γ (IFNγ), which deactivates the host enzyme indoleamine 2,3-dioxygenase (IDO), leading to the degradation of the host tryptophan. This process increases *Mtb’s* reliance on its tryptophan biosynthetic machinery. Building on this structural mimetic of tryptophan, intermediates were made and found effective against *Mtb* both in vitro and in mice [168]. Many research groups have utilized in silico screening to identify inhibitors of indole-3-glycerol-phosphate synthase (TrpC), resulting in the discovery of a competitive inhibitor, ATB107, which demonstrates activity against *Mtb* in axenic cultures and inside macrophages [170]. Interestingly, the effect of ATB107 is not reversed by the addition of tryptophan, suggesting potential targets outside the tryptophan biosynthesis pathway. This underscores the challenges associated with target-based drug discovery methods and whole-cell approaches, as hit compounds often exhibit multiple targets.

The discovery of drugs for treating TB targeting metabolic enzymes has drawn insights from various fields. One example is the development of antimicrobial agents inspired by the herbicide sulphometuron methyl (SMM) [171], known for inhibiting branched-chain amino acid synthesis in plants. SMM has been found effective against acetohydroxyacid synthase (IlvB1), the primary enzyme in *Mtb*’s branched-chain amino acid biosynthesis. IlvB1 is crucial in the absence of supplemented amino acids, and deleting it leads to the death of *Mtb* in vitro and attenuation of infection in mice models. Chemically validating this target, SMM proves potent against *Mtb* in vitro and hinders its growth in the lungs of infected mice.

### 5.4. Fatty Acid Metabolism

Pantothenate or vitamin B5 is an important cofactor for CoA and the attenuating mutation *∆panCD* deletion renders *Mtb* auxotrophic in producing this essential cofactor CoA. This has attracted many researchers to inhibiting the *Mtb*’s pantothenate synthase (PanC) and pantothenate kinase (PanK) with the help of different drugs in efforts to control its growth. But this was met with limited success: studies later revealed that it requires more than 95% efficiency to effectively knock down this gene to suppress *Mtb* growth [172,173]. Through hypomorph mutation studies, it was found that by targeting the *coaBC* gene instead of *panB*, *panC*, and *coaE*, which are involved in CoA biosynthesis, effective *Mtb* attenuation could be achieved. The *coaBC* silencing induced a bactericidal phenotype, whereas silencing the other genes had a bacteriostatic phenotype [173]. This led to utilizing *coaBC* as a metabolic target to attenuate *Mtb* growth. Similarly, aspartate decarboxylase (PanD), which catalyzes the conversion of aspartate into beta-alanine, was found to be inhibited by pyrazionic acid [174]. The active molecule binds to PanD and inhibits CoA biosynthesis. CoA is an important molecule in *Mtb* since CoA acts as an important cofactor for many other metabolic enzymes during stress. The CoA is an important cofactor for ACPs (acyl carrier proteins) in *Mtb*. ACPs are involved in the generation of mycolic acid, mycobactins, lipids, and other vitally important cellular functions. The depletion of CoA through various drugs leads to failure of the transfer of the 4′-phosphopantetheine (Ppt) group of CoA to ACPs through two transferases, PptT and AcpS. PptT can be effectively inhibited by an amidino-urea inhibitor, which results in the generation of only holo-ACPs devoid of cellular functions [175]. Alternatively, PptH is a hydrolase found in *Mtb* function as an antagonist of PptT. PptH and the amidino-urea inhibitor work synergistically and were found to inhibit holo-ACPs.

### 5.5. Posttranslational Metabolism

The adaptation of *Mtb* to various nutritional stresses under in vivo conditions also leads to various resistance mechanisms. During in vivo nutritional stress, many hyperphosphorylated guanine nucleotides are synthesized (e.g., ppGpp and pppGpp). A bifunctional protein known as Rel catalyzes the hydrolysis and synthesis of these hyperphosphorylated nucleotides [176]. The inactivation of *rel* had pleiotropic effects that affected the survivability of the *Mtb* under stress and antibiotics [177]. The silencing of *rel* increases the efficacy of first-line *Mtb* drugs under nutrient-rich conditions in in vitro models [178].

### 5.6. Energy Biosynthesis

ATP (adenosine triphosphate) homeostasis is a key regulator of bacterial survival in host cells under both in vitro and in vivo conditions. The generation and maintenance of ATP under nutrient-deprived and hypoxic conditions are important for MDR-TB and XDR-TB. The ETC (electron transport chain) plays a crucial role in the generation of a proton gradient across the membrane, which, in turn, leads to the generation of the ATP. Enzymes like NADH dehydrogenases (NDH-1 and NDH-2) and succinate dehydrogenase (SDH) transfer electrons to MK (menaquinone), a lipid-soluble electron carrier. MK then transfers electrons to various components such as nitrate reductase, fumarate reductase, the cytochrome bc1–aa3 complex, or cytochrome bd oxidase [179,180]. BDQ is an inhibitor that exhibits bactericidal activity against *Mtb* in vitro in mice [181]. The *Mtb* develops resistance to this drug according to a mutation in *aptE,* which is a component of the F_0_ subunit of the ATP synthase. BDQ along with other drugs is currently being evaluated in ongoing trials for its role in shortening the treatment duration for XDR-TB and drug-sensitive infections, showing promising preliminary results [182,183]. There are many new inhibitors developed against enzymes upstream of ATP synthase, such as Q203, which inhibits the cytochrome-b subunit (QcrB, Rv2196) of the cytochrome bc1, transferring electrons from MK to cytochrome c oxidase and thus halting ATP generation. Q203 is bacteriostatic in vitro but not against non-replicating *Mtb* [184]. Genetic disruption of the cytochrome bd oxidase, encoded by *cydAB*, synergizes with Q203, resulting in a bactericidal response against replicating and non-replicating *Mtb* [184].

Delamanid and pretomanid belong to a class of nitroinidazole drugs that is used for the treatment of drug-resistant non-replicating *Mtb* [185]. Both these compounds require the deazaflavin-dependent nitroreductase (Ddn) protein encoded by the *ddn* gene, which is an F420-dependent nitroreductase [185,186]. The Ddn enzyme converts the delamanid and pretomanid into metabolites that generate nitric oxide (NO). The NO generated reacts with the cytochrome c oxidase and inhibits the ETC and inhibit non-replicating *Mtb* [186,187]. Protemanid, along with other drugs like bedaquline or oxazolidinones, is used and has shown effective results against drug-resistant *Mtb* (Nix-TB) in mice models.

### 5.7. Alternative Carbon Metabolism

While glycerol is commonly employed as a carbon source for in vitro axenic culture to grow *Mtb*, it holds little relevance in vivo, where *Mtb* primarily relies on fatty acids as its carbon source [188,189]. The glucose metabolism is interconnected with the glycerol metabolism through dihydroxyacetone phosphate and is crucial for the survival of *Mtb* in mice. *Mtb* is not vulnerable to inhibitors dependent on the glycerol metabolism but may respond to inhibitors targeting alternative carbon metabolism pathways. In the absence of carbohydrates, most microorganisms, including *Mtb*, utilize the glyoxylate cycle for growth on fatty acids or acetate.

In *Mtb*, isocitrate lyase (ICL) is a bifunctional enzyme that converts isocitrate into glyoxylate and succinate. Glyoxylate is a common carbohydrate source in microorganisms in the absence of common carbohydrates as a source of energy [190,191,192]. ICL plays a crucial role in both the glyoxylate pathway and the methylcitrate pathway. ICL knockout leads to the accumulation of toxic metabolites and essentially affects *Mtb*’s survival. Since humans lack the ICL homologs, it becomes a key target for the development of inhibitors to control *Mtb*’s growth. 3-NP (3-nitro propionate) is an inhibitor known to inhibit ICL in other organisms [192] and was found to be active against *Mtb* growth on fatty acids and in macrophages [189]. 3NP not only targets ICL but also has bactericidal effects on both wild-type *Mtb* and ICL knockouts, suggesting broader targets in *Mtb*. 3-NP has also shown inhibitory activity against succinate dehydrogenase (SDH), a TCA cycle enzyme, possibly contributing to its activity against ICL knockouts.

Another enzyme, malate synthase (GlcB), that converts glyoxylate into malate is utilized by *Mtb* during carbohydrate-deprived conditions to utilize fatty acids as an alternative carbon source. Unlike ICL, researchers have developed many distinct inhibitors through target-based approaches in GlcB. Many phenyl diketo acid derivatives have been shown to inhibit GlcB in vitro and have demonstrated activity against entire *Mtb* cells grown on acetate and in low-oxygen conditions [193]. Additional evidence supporting GlcB as a novel drug target includes its essential role in *Mtb* survival during the chronic phase of mouse infection, as well as its necessity for glyoxylate detoxification and resistance to fatty-acid-associated toxicity [194].

### 5.8. Iron Metabolism

Iron availability is key for *Mtb* pathogenesis in vivo and has garnered attention for the development of new antibiotics against the pathogen [195,196]. *Mtb* scavenges iron from its host through two siderophores: mycobactin, which is a cell wall-associated protein, and carboxymycobactin, which is secreted for scavenging. There are many enzymes involved in the iron-chelating pathway. MbtI initiates the synthesis of salicylate from chorismate, followed by activation by MbtA. The activated salicylate then binds covalently to the carrier protein domain of MbtB [197]. Subsequently, MbtB combines the salicylate with L-threonine, initiating cyclization of the intermediate. Progressing through the synthetic pathway, MbtE condenses the molecule with modified L-lysine, MbtD attaches a β-keto group, and finally, MbtF combines the molecule with another modified lysine, resulting in the release of mycobactin [197]. Highlighting the potential of this pathway as a drug target, the growth attenuation of an *mbtE* deletion strain in vitro was observed, a condition reversed by iron and exogenous siderophores [198]. Expanding on the understanding of siderophore biosynthesis mechanisms, various research groups have employed rational design to create inhibitors for mycobactin biosynthetic enzymes. The initial compound, 5′-O-[N-salicyl-sulfamoyl adenosine] (salicyl-AMS), was crafted to mimic an intermediate in the MbtA reaction [199]. Salicyl-AMS effectively inhibits MbtA in vitro and restrains *Mtb*’s growth in iron-depleted conditions. It has demonstrated some activity in a murine model of *Mtb* infection, serving as in vivo chemical evidence for this new anti-tubercular target [195].

## 6. Challenges in Targeting the Metabolic Rewiring in *Mtb*

The key bottleneck in developing new drugs for targeting the metabolic rewiring in *Mtb* is our limited understanding of *Mtb*’s metabolism in human hosts. Despite insights from laboratory studies revealing the bacteria’s metabolic flexibility, aspects like alternative pathways, metabolite accessibility, and regulation during host infection remain unclear. The discovery of potent drug targets often comes from genetic inhibition studies, assessing the impact of genetic knockdown on *Mtb*’s survival in vitro, in macrophages, and in mouse models. While such relative studies aid target prioritization, they may not accurately represent chemical inhibition, especially for irreversible or conformational-changing inhibitors. Variances in the target vulnerability between in vitro and in vivo conditions or model organisms underscore the limitations of these approximations in understanding human disease.

The biological and ethical limitations of carrying out infection studies in human lungs have restricted researchers from relying on mainly autopsy studies, biopsies, or lung resections to understand the disease physiology. Clinical samples are constrained in availability, transportation, and safety guidelines, and give only temporal insight into the disease progression. While the bacteria obtained from sputum or aerosols are more accessible alternatives to tissue resection for studying microbial physiology, they are distal to the disease locus [200]. To directly examine the microbial state within the host, it is crucial to employ culture-independent analytic techniques with near-single-cell sensitivity. The imminent application of single-cell transcriptomic approaches to mycobacteria holds the promise of offering valuable insights into the mycobacterial state during infection and the interaction with the host. However, a challenge lies in the limited correlation between transcriptional profiles and metabolic states. A more promising approach involves adapting single-cell metabolomic techniques, commonly used to study bacterial heterogeneity, to sputum-derived bacilli [201]. This adaptation aims to provide qualitative information about the metabolic status, albeit for a limited number of metabolites.

The advancement of chemical probes, derived synthetically and functionalized from known metabolites, provides an important toolset for investigating *Mtb*’s physiology. These probes can be used to target and label specific pathways, used for dissecting the disease etiology and drug targets [201,202]. By incorporating fluorescent or radio labels, these probes could enable the direct visualization of metabolite incorporation into specific pathways and bacterial metabolic activity in host tissue samples. This approach has the potential to complement existing methods, such as imaging using radiolabeled glucose, by providing a novel and convergence of in situ techniques of imaging, metabolomics, and sequencing. This multimodal analytical approach is useful in gaining direct insights into disease biology.

Despite these limitations, comprehensive metabolite profiles from clinical samples or non-human primates would greatly aid in developing improved models to evaluate the effectiveness of metabolism-targeting antitubercular drugs. The current models should accurately reflect the metabolite environment of infection during various stages and the various metabolic states adopted by *Mtb*. However, the simultaneous, absolute, and non-targeted quantification of metabolites using mass spectrometry is technically challenging. Nevertheless, targeted approaches to metabolite quantitation, particularly in the search for diagnostic biomarkers, offer a feasible opportunity to construct higher-resolution metabolic landscapes of TB disease pathology.

It is also essential to assess the biochemical aspects to identify the most chemically vulnerable and tractable targets in the metabolic pathway. According to classic kinetics, drug design should target the rate-determining enzyme of a pathway, as inhibiting the slowest step maximally impacts the overall pathway flux [203]. However, challenges arise in determining which enzymes are rate-determining, assessing the active site pocket depth for inhibitor identification, and predicting the outcome of inhibiting these steps on the bacterial viability. Chemical optimization should be taken into account in drug activity, crucial for correlating the in vitro and in vivo activity, especially considering the differences between closed in vitro and open in vivo systems [204].

With the advent of new methodologies and techniques that can be used to study in situ infection and mimic a similar environment as in vivo diseased conditions, we can contribute to a greater understanding of the pathophysiology of *Mtb* infection and understand and utilize its vulnerability. Understanding the key pathways of the bacteria can reveal new targets but it is also imperative to identify targets that can be targeted easily. Advances in our chemical and biological understanding of the *Mtb* pathway will lead to the enhanced development of drugs with better drug targeting, increased cell penetration, and improved pharmacokinetics.

## 7. Conclusions

To achieve the end TB goals, we must develop new vaccines, along with novel therapeutic and diagnostic methods. For this, we must have a clear understanding of the dynamic interaction between the pathogen’s metabolism and anti-TB drugs’ pharmacological pressure. In this review article, we have discussed several critical insights into the metabolic remodeling of *Mtb* in response to antibiotics and drugs, as well as under antibiotic-resistant conditions. Firstly, the inherent nature of the metabolic flexibility of *Mtb* enables it to survive and adapt in an antimicrobial milieu. *Mtb* adapting to these unfavorable conditions involves metabolic rewiring and a dynamic shift in the biochemical pathways and nutrient metabolism that is crucial for the pathogen’s survival and persistence. The modification of the lipid metabolism, redox homeostasis, and energy production pathways are extremely critical, reflecting *Mtb*’s strategic response to drug-induced stress. To combat the emergence of MDR-TB and XDR-TB strains, we urgently need a novel therapeutic strategy. Our review suggests that targeting the genes or pathways essential for metabolic adaptations of *Mtb*, especially those unique to its response to specific drugs, could be a promising approach. Moreover, we emphasize the importance of understanding the intrinsic and acquired mechanisms of drug resistance in *Mtb* using various efflux pumps, genetic mutations, or modifications of the cell envelope. We anticipate that the existing drugs can be repositioned and repurposed, as well as novel drugs being developed to target specific metabolic pathways within *Mtb*. However, the intrinsic characteristics of *Mtb*, such as a thick cell wall and unique metabolic pathways, pose significant challenges. Developing effective therapeutic strategies requires advances in our understanding of *Mtb*’s metabolism, particularly in the context of the host environment.

Different strategies are used to combat the intrinsic and acquired resistance in *Mtb*: for example, (a) efflux pump inhibitors are being investigated to prevent drug expulsion and increase intracellular drug concentrations, as well as a combination therapy that pairs current medications with adjuvants to increase the efficacy or neutralize resistance. (b) It is important to evaluate the potential of host-directed therapies for their capability to modulate the immune response to achieve better bacterial eradication. (c) Developing novel drugs that target unexplored bacterial pathways may contribute to reducing cross-resistance with existing antibiotics. (d) It may be possible to identify molecular targets for new interventions using genetic mapping, while bacteriophages can be used to target resistant *Mtb* strains. (e) Furthermore, rapid diagnostics can help identify resistant strains early and provide timely and appropriate treatment adjustments. However, we should explore each of these strategies in greater detail to expand our weapons against TB resistance.

## Figures and Tables

**Table 1:** Examples of anti-TB drugs based on administration protocols.

Lines	Grouping	Drugs	Common Side Effects	References
First-line	Group 1 (oral)	Isoniazid	Hepatoxicity, neuropathy	[2]
		Rifampicin Rifapentine Rifabutin	Hepatotoxicity, thrombocytopenia, neutropenia, induction of cypP450	[3,4]
		Pyrazinamide	Hepatotoxicity, hyperuricemia	[4,5]
		Ethambutol	Optic neuropathy, hepatotoxicity	[6]
Second-line	Group 2 (injectable)	Aminoglycosides: Streptomycin Kanamycin Amikacin	Ototoxicity, nephrotoxicity	[7,8,9]
		Polypeptides: Capreomycin Viomycin	Hyperaldosteronemia, renal tubular dysfunction	[10,11]
	Group 3 (oral and injectable)	Fluoroquinolones: Ciprofloxacin Ofloxacin Levofloxacin Moxifloxacin Gatifloxacin	Tendonitis arthropathy psychiatric disturbance increases in transaminases. Nausea, diarrhea, headache, and dizziness	[12,13,14,15,16]
	Group 4 (oral)	Para-aminosalicylic acid Cycloserine Terizidone Ethionamide Prothionamide Thioacetazone Linezolid	Diarrhea, hypothyroidism, goiters, drowsiness, anxiety, mood disturbance, psychosis, and seizures Low gastrointestinal tolerability Gynecomastia Peripheral and ocular neuropathy, anemia thrombocytopenia, hyperlactatemia, diarrhea, nausea, hypoglycemia reticulocytopenia	[12,17]
Third-line	Group 5 (oral and injectable)	Clofazimine Amoxicillin + clavulanate Imipenem + cilastatin Clarithromycin	Gastrointestinal obstruction and bleeding Liver injury, pancreatitis Seizures Nausea, diarrhea, and headache	[12,18,19,20]

**Table 2:** Anti-TB drugs and their mode of action [21].

Drug Classification	Class of Drug	Anti-TB Drug	Mechanism of Action	Mechanism of Resistance	References
Cell envelope synthesis inhibitor	Peptidoglycan	Cycloserine	Inhibits D-alanine residue-forming enzymes	Mutations in *alrA*	[22]
		Terizidone	Cycloserine derivative		[23]
		Capreomycin	Peptidoglycan breakdown	Mutation in *tlyA*	[24]
	Arabinogalactan	Ethambutol	Inhibits arabinosyltransferase, arabinose acceptor	*embCAB* operon	[25,26]
	Mycolic acid	Isoniazid	Activates the *katG* enzyme and inhibits the *inhA* gene	Mutations in *katG* and inhA genes	[27,28]
		Triclosan	Inhibits the *inhA* enzyme	–	[29,30]
		Pyridomycin		Mutations in *inhA*	[31]
		Ethionamide		Mutations in *inhA*, *ethA*	[32]
		Prothionamide		Mutations in *ethA*	[33]
		Delamanid	Nitric acid release by the Ddn enzyme		[34,35]
		SQ109	Membrane transporter MmpL3		[36]
	Lipid biosynthesis	Thiocarlide	Inhibits oleic acid synthesis	Mutations in *ethA*	[37]
Protein synthesis inhibitor	Aminoglycosides	Streptomycin	Binds to the 30S subunit of the ribosome	Mutation in *rpsl*	[38]
		Amikacin		Mutation in *rrs*	[39]
		Kanamycin			
	Oxazolidone	Linezolid	Binds to the 50S subunit of the ribosome	Mutation in G2576T(23S)	[40]
		Sutezolid			[41]
Nucleic acid inhibitor	Rifamycins	Rifampicin	RNA polymerase inhibitor	Mutations in the *rpoB* gene	[42,43]
		Rifapentine			[44]
		Rifabutin			[45]
		Rifalazil			[46]
	Para-aminosalicylic acid	PAS	Folic acid synthesis inhibitor	Mutations in *thyA*	[22]
	Quinolones	Levofloxacin	DNA gyrase inhibitor	Mutations in *gyrA*	[47]
		Moxifloxacin			
Other		Pyrzinamide	Interferes with binding to mRNA	Mutations in *rpsA*, *pncA*	[48,49]
New drugs		Bedaquiline	Inhibits ATP synthase enzyme	Mutations in the *atpE* gene	[50,51]
		Pretomanid	Inhibits the synthesis of mycolic acids	Mutations in *ddn*, *fgd1*, *fbiA*, *fbiB*, *fbiC*, and *fbiD*	[52]
		Clofazimine	Inhibits DNA replication	Mutations in *Rv0678*	[53]

**Table 3:** Repurposed drugs to combat *Mtb* infection.

Class	Drugs	Original Target/Function	References
Antibiotics	Biapenem	Against *P. aeruginosa* strains	[55]
	Tebipenem	Otitis	[55]
	Clofazimine	Leprosy	[56]
	Linezolids	Broad spectrum against Gram-positive bacteria	[57]
Non-steroidal anti-inflammatory	Diminazene	Trypanosomiasis and babesiosis	[58]
	Ebselen	Anti-inflammatory and antioxidant	[59]
Anti-parasitic	Artemisinin	Anti-malarial	[60]
	Mefloquine	Anti-malarial and anti-leishmania	[61,62]
	Pyronaridine	Anti-malarial	[63]
Antiviral	Isoprinosine	Subacute sclerosing panencephalitis	[64]
Anticancer	Bortezomib	Multiple myeloma	[65]
	Elesclomol	Metastatic melanoma	[66]
	Bis-biguanide dihydrochloride	HIV and the immobilization of sperm	[67,68]
Cardiovascular drugs	Verapamil	Prevents migraines and headaches	[69,70]
	Simvastatin	Reduces stroke in hyperlipidemic patients	[71]
	Pravastatin	Treats hypocholesterolemia	[72]
Other	Metformin	Type II diabetes	[73]
	Rimonabant	Obesity	[67]
	Pranlukast	Bronchoconstriction	[74]
	Cyclosporin A	Immunosuppressant	[75]

**Table 4:** Anti-mycobacterial drugs and their effects on *Mtb* metabolic pathways during infection.

Treatment Regimen	Name of Drugs	Drug Metabolism	Modulation in Metabolic Pathways	Refs.
For treating drug-susceptible TB	Isoniazid (INH)	Prodrug activated by *Mtb* catalase-peroxidase (*katG*)	Alters lipid metabolism and redox homeostasis, decreases ROS levels, metabolic activity, and SigB/SigE responses	[76]
	Rifampicin (RIF)	Metabolizes in the human liver	Induces mistranslation of *rpoB*, reduces replication, and alters metabolic pathways associated with pyrimidine, purine, arginine, phenylalanine, tyrosine, and tryptophan synthesis, along with TCA, efflux pumps, and phosphoenolpyruvate metabolism	[43,78,79,80,81,82,83,84]
	Pyrazinamide (PZA)	Prodrug activated by *Mtb* nicotinamide (PncA)	Alters TCA pathways and energy production, leading to adaptive responses in *Mtb*, influencing gene expression and metabolic pathways, which need further exploration	[49,85,86,87]
	Ethambutol (EMB)	Ester-protected ethambutol (prodrug) activated by mycobacterial hydrolases	Disruption of cell wall components can lead to various metabolic changes (e.g., SigB or SigE sigma factors) that are an area of ongoing research	[25,88,89,90,91,92,93]
For treating MDR-TB and XDR-TB	Fluoroquinolones (e.g., moxifloxacin)	Undergo phase I and II metabolic reactions in the human liver	Inhibition of DNA-related processes may influence nucleotide metabolism and energy production pathways	[86,94,95]
	Cycloserine	Metabolizes in the human liver	Pathways regulating the cellular integrity and amino acid metabolism may be modulated. Future studies, employing transcriptomics and metabolomics, might unravel further insight into this area	[96,97,98]
	Bedaquiline (BDQ)	Metabolized by human Cytochrome P450 isozyme 3A4 (CYP3A4) into a less active metabolite	Several stress proteins and transcription factors (including SigG, *Rv0324*, and *Rv0880*) are upregulated upon inducing stress	[99,100,101,102,103]
	Ethionamide (ETH)	Prodrug activated by *Mtb* monooxygenases (EthA)	Induced response is like INH stress with genes involved in lipid metabolism and cell wall synthesis	[86,104,105,106]
	Aminoglycosides (e.g., Amikacin)	Mostly remain unchanged	Alters genes involved in drug efflux (tap) and ribosomal protection (e.g., *eis*) to reduce sensitivity toward amikacin stress	[107,108,109,110,111]

## Data Availability

Not applicable.

## Abbreviations

TB; *Mtb*, Mycobacterium tuberculosis; FLDs, First-line drugs; SLDs, Second-line drugs; STR, Streptomycin; INH, Isoniazid; PZA, Pyrazinamide; RIF, Rifampicin; FQ, Fluoroquinolone; AMK, Amikacin; KAN, Kanamycin; CAP, Capreomycin; RFB, Rifabutin; CLO, Clofazimine; CLR, Clarithromycin; THZ, Thiacetazone; BDQ, Bedaquiline; DMD, Delamanid; EMB, Ethambutol; POA, Pyrazinoic acid; VIM, Viomycin; LZD, Linezolid; SZD, Sutezolid; DCS, D-cycloserine; PRM, Pretomanid; BTZ, Benzothiazinone; ETH, Ethionamide; PAS, Para-aminosalicylic acid; MDR-TB, Multidrug-resistant tuberculosis; XDR-TB, Extensively drug-resistant tuberculosis; TDR-TB, Totally drug-resistant tuberculosis; HIV, Human immunodeficiency virus; WHO, World Health Organization.
